# Setting Up a Local Registry to Improve the Care of Patients With Primary Biliary Cholangitis

**DOI:** 10.7759/cureus.25247

**Published:** 2022-05-23

**Authors:** Laith Alrubaiy, Ceyhun A Oztumer

**Affiliations:** 1 Gastroenterology, St. Mark's Hospital, London, GBR; 2 Gastroenterology and Hepatology, St. Mark's Hospital, London, GBR

**Keywords:** treatment guidelines, registry, hepatology, quality improvement project, primary biliary cholangitis

## Abstract

Primary biliary cholangitis (PBC) is a rare but progressive chronic disease of the liver. The national guidelines aim to standardise the care of patients with PBC across the UK. The guidelines also recommend routine screening for the presence of symptoms in patients with PBC, although none suggest how such screening should be achieved in clinical practice. We aim to develop a sustainable and comprehensive local registry for patients with PBC to examine current practice and help define long-term complications and survival.

Setting up the registry involves working with several workstreams to identify the data required for the registry, technical IT infrastructure to support the data collection, and a steering committee to oversee the work of the PBC registry. This registry will involve patients aged ≥18 years from the London North West University Health Trust hospitals with a diagnosis of PBC as defined by the British Society of Gastroenterology (BSG) and the European Association for the Study of the Liver (EASL) criteria. Patients will not be subjected to any additional treatments or investigations as the registry will be part of routine clinical practice.

## Introduction

Primary biliary cholangitis (PBC) is a chronic cholestatic liver disease. Typical PBC-related symptoms include fatigue, pruritus, dry eyes and mouth, and occasional abdominal and bone pain. Fatigue can be profound and persistent and is typically unrelated to the histological stage or activity of the disease [1,2].

Recent years have seen a significant evolution in the treatment of PBC [3-6]. Furthermore, the importance of health-related quality of life (HRQoL) is widely appreciated, and approaches to the management of the symptoms contributing to HRQoL, most notably pruritus, continue to improve [7,8]. HRQoL provides insights into how patients perceive their disease processes, which may be different from how clinicians perceive them [9]. In light of the importance of symptoms and their impact on patients’ quality of life, all current guidelines, including those from the British Society of Gastroenterology (BSG) and the European Association for the Study of the Liver (EASL), recommend routine screening for the presence of symptoms in PBC [1,3]. However, none of these guidelines suggest how such screening should be achieved in practice, and at present, there are no validated tools suitable for screening use in practice.

The PBC-40 questionnaire is a patient-derived, disease-specific HRQoL measure validated for self-completion by PBC patients [10]. Although PBC-40 is a valuable research tool, including as a patient-reported outcome measure in trials of new therapies, it is too lengthy and time-consuming to use in routine clinical practice. To make the collection of HRQoL a routine clinical practice, the PBC-10, which is a short version of the PBC-40 questionnaire was developed and it can be self-completed by patients in about five minutes [7].

A recent study has shown gaps in the quality of care delivered to patients with PBC [11]. Several factors may affect the delivery of optimal treatment to patients with PBC. Most specialist hepatologists are clustered at liver transplant centres and tertiary hospitals [12]. Therefore, the majority of patients with PBC are managed by primary care physicians, general gastroenterologists, or acute medical physicians [11].

This study aims to develop a sustainable, comprehensive, local registry for PBC that is professionally supported and managed. Data collected is extremely valuable to re-define the impact of PBC on HRQoL and disease progression, as well as to evaluate the heterogeneity in the use of current therapies, variation in healthcare delivery, cost of therapy, disease outcomes, and mortality. Data collected from the registry will be used to examine the impact of PBC on clinical presentation, HRQoL, complications, and mortality. Registry data will also help support the clinical decisions, examine the use of health resources, and identify the healthcare costs of managing patients with PBC. Anonymised aggregate data will allow benchmarking of patients’ outcomes compared to other units.

## Materials and methods

We included discussions with hepatologists, patients, IT specialists, and clinicians who look after patients with PBC. We divided the discussions into several workstreams to develop the registry and meet the requirements. They include data collection workstreams to identify data needed for the registry that can meet the national guidelines and support patient care; technical IT infrastructure workstreams to develop the IT software needed for the registry, this has to be compatible with current clinical software used in the hospital and be a part of routine clinical care; steering committee workstream to oversee the work of the PBC registry and ensure all clinical governance aspects are met.

## Results

The outlines of the PBC patients registry were developed and agreed upon. The plan is to implement the registry in Northwest London NHS trust and roll it over to other hospitals. The registry will include adult patients with a diagnosis of PBC.

Data collection

We aim to prospectively collect data from patients with PBC over a three-year period from the London North West University Health Trust (St Mark’s, Northwick Park, Central Middlesex, and Ealing Hospitals). Patient treatment will not be affected in any way. Only “routine care” data will be collected and patients will not be subjected to any additional examination for the registry/study; the only exception is asking patients to complete the PBC-10 quality of life (QoL) questionnaire, but these are non-invasive and would not affect routine clinical care. Data will be collected using two reporting forms.

1. Case report form: the baseline dataset at enrolment, incorporating all relevant variables.

2. Follow-up review form: follow-up data will be collected at 3, 6, and 12 months, signifying changes in medications, complications, hospital admissions, and survival status. If the patient undergoes a liver transplant or dies, their data will be recorded at that date.

The dataset will be sufficiently robust to provide a comprehensive overview of all aspects of PBC care within North West London. The initial dataset was established following consultation with our medical and specialists’ collaborators. Harmonised data will be collected using an online platform to include informed consent, patients' demographic data (date of birth, sex, address, identifiable ID), as well as disease and clinical data (date of diagnosis of PBC, and blood tests including renal function, liver function, full blood count, clotting profile for every visit or review). If cirrhosis is present, the Model for End-Stage Liver Disease (MELD) and Child-Pugh scores will be captured. The registry will also include data on the quality of life measures using SF-12 and the short version of PBC-QoL (PBC-10) and dates of key investigations (liver biopsy, autoantibodies, imaging, etc.).

IT Infrastructure

This workstream identified the technical infrastructure to house the registry, and use an established IT platform to capture the relevant data. This online platform will be supported by the NHS IT department. It has been chosen because it already meets key criteria, as required by the regulatory authorities. The IT software will be a cloud-based electronic data capture platform that easily enables easy capture of high-quality, reusable data suitable for registry setup. In line with patients' confidentiality, the data will be held in anonymised form in a remotely-hosted, professionally-managed, secure data warehouse. This will be fully compliant with Good Clinical Practice (GCP) [13] and certified in the field of information security (Standards for Information Security Assurance). The IT structure will provide an opportunity to collect a full audit trail of the data and to import and export compatible file types.

Governance and steering committee

A steering committee in consultation with other gastroenterologists and hepatologist colleagues will lead and oversee the work of the PBC registry, including budget management, the monitoring and management of associated timescales, and ensuring that appropriate quality measures are in place.

Patient involvement

Members of patient charities and PBC-UK will be consulted about the registry to ensure appropriate patient representation. The registry and relevant dataset were developed based on the review and feedback from our patient advisory group. A discussion with the current patient group has confirmed their support for this initiative.

## Discussion

A patient registry is defined as an organised system that uses observational methods to collect uniform data on a patient population defined by a particular disease, exposure, or condition, which is followed over time. We plan to develop and implement a local registry for patients with PBC. This registry will provide an opportunity to identify new models for quality improvement. The patient registry will provide up-to-date “real-time” data on patients with PBC to examine current practice, develop new scoring systems, and define long-term complications and survival. There are also opportunities to use registry data to support future registry-based studies. 

The PBC registry will benefit relevant stakeholders in different ways. For hepatologists, it will collect data about patients with PBC, their clinical presentation, complications, prognostic factors, current treatment practices, and outcomes. The registry can be used in clinical audits to assess the adherence to evidence-based guidelines and to identify specific aspects of patients with PBC that might otherwise be overlooked. Patients and patient charities will also benefit from the registry by increasing their understanding of the natural history of a disease and getting a better picture of local and national performance. Importantly, the data acquired may allow modification of the liver allocation strategy, such that these patients can be transplanted earlier. The accurate costs associated with the management of patients with PBC are unknown. Financially, the data acquired from the registry will provide a realistic estimate of the healthcare costs associated with the management of these patients. 

## Conclusions

This project helped to set up the first national registry for patients with PBC. Although the national UK-PBC audit has identified gaps in the care for patients with PBC, we hope that this registry setup will be used as a quality improvement tool to improve the care of patients. Future data collection will show its effectiveness and usefulness.
